# Is Total Knee Replacement Justified in the Morbidly Obese? A Systematic Review

**DOI:** 10.7759/cureus.804

**Published:** 2016-09-23

**Authors:** Raju Vaishya, Vipul Vijay, David Wamae, Amit Kumar Agarwal

**Affiliations:** 1 Orthopaedics, Indraprastha Apollo Hospitals

**Keywords:** total knee replacement, assessment, outcomes, complication, morbid obesity, systematic review

## Abstract

Total knee replacement (TKR) comprises a significant, growing aspect in the management of patients with advanced arthritis of the knee for which conservative medical therapy has failed. Obesity, a rising epidemic, is considered an important independent risk factor in the development of osteoarthritis (OA). An aging population and increasing incidence of obesity contribute to a higher prevalence of OA and a subsequent greater need for TKR. The numbers of morbidly obese (MO) people undergoing TKR has consistently been rising. However, there have been concerns among patients and surgeons about the outcomes and complications of TKR in MO patients, especially given the morbidities associated with obesity. The goal of this systematic review was to assess relevant, up-to-date data on the safety, outcomes, and complications associated with TKR in MO patients.

## Introduction

Total knee replacement (TKR) constitutes a significant development in the management of patients with advanced arthritis of the knee for which conservative medical therapy has failed. The number of TKRs done worldwide continues to rise annually because, arguably, it is one of the most successful orthopedic procedures resulting in a substantial and sustained improvement in pain and disability of the knee [1]. Annually, it is affirmed that about a million TKRs are performed in the world [1-2]. The increasing demand for TKR is as a result of several well-documented reasons, and the procedure is beneficial and improves the quality of life for people with severe osteoarthritis (OA) of the knee [3-4].

Obesity is a global problem and affects nearly a third of the population in the United States and the United Kingdom [2]. It is a rising epidemic and one of the major public health problems [2]. Obesity is considered an important independent risk factor in the development of OA [4-7]. In a previous report, the World Health Organization (WHO) stated that 500 million of the world's population were obese [2]. Both the aging population and the increase in obesity contribute to a higher prevalence of OA and a proportionally greater need for TKR [3]. In the literature, primary TKR is more widespread among patients who are obese compared to patients who are considered to have a normal body mass index (BMI) [7]. The demand for TKR is projected to rise in the future, and it seems to parallel the increase in obesity in America [8-10]. The numbers of morbidly obese (MO) people undergoing TKR has consistently been rising according to studies from 1990 to 2005 [11]. However, there have been concerns among patients and surgeons about the outcomes and complications of TKR in MO patients. It is partly due to the fact that obesity has been associated with morbidities, such as Type 2 diabetes mellitus, hypertension, coronary artery disease, and cancer, in addition to OA [8].

This systematic review was aimed to assess relevant, up-to-date data on the safety, outcomes, and complications associated with TKR in MO patients. 

## Materials and methods

Our search strategy was executed according to the recommendations of the Cochrane group. We completed an electronic database search of the English literature on Medline, EMBASE, CINAHL, Cochrane Central Register of Controlled Trials (CENTRAL), DARE, and other databases for relevant articles using the following MeSH terms and keywords: human studies, complications, body mass index (BMI), and functional scores. The only limits applied to the search were that studies had to be published in English by December 18, 2015. The search was conducted from December 18-24, 2015.

The lists of references of the retrieved publications were checked manually. It included a search for additional articles which met the inclusion criteria but were not picked up by the electronic search. Two researchers (DW and VV) individually reviewed the literature to identify relevant articles for further review and to avoid inclusion of duplicate articles.

Using the criteria mentioned above, the articles for further inclusion in the study were independently selected by the reviewers. In case there were differences of opinion between the two observers, the disagreement was resolved by taking the view of the senior author (RV). Studies were included if they met all the inclusion criteria and were not blinded regarding the source, affiliation, or author name. We included all the studies which used cemented as well as uncemented total knee prostheses. We excluded review articles, expert opinions, surgical techniques, and abstracts from scientific meetings. Articles in this review were assessed for the level of evidence. Prospective, case-control, and retrospective cohort studies with evidence level II were included. We included studies that clearly described the MO population from the control (non-morbid obese (NMO)) group according to the WHO guidelines. Other studies that used BMI as a definition for obesity but did not use the clearly defined WHO groupings were also excluded. The present systematic review followed the Preferred Reporting Items for Systematic Reviews and Meta-Analyses (PRISMA) guidelines. The primary research question in the present systematic review was to study the outcome of primary TKR in the morbidly obese population. As a measure of results, the studies were assessed for functional recovery (using standardized knee scores) and complications (infection, deep vein thrombosis (DVT), radiolucencies, and mortality). The studies were also evaluated for revision rates and overall implant survivorship.

The studies included in this systematic review were assessed for BMI, change in Knee SocietyScore (KSS), percentage incidence of superficial infection, percentage incidence of deep infection, as well as overall complication rates.

## Results

**Figure 1:**
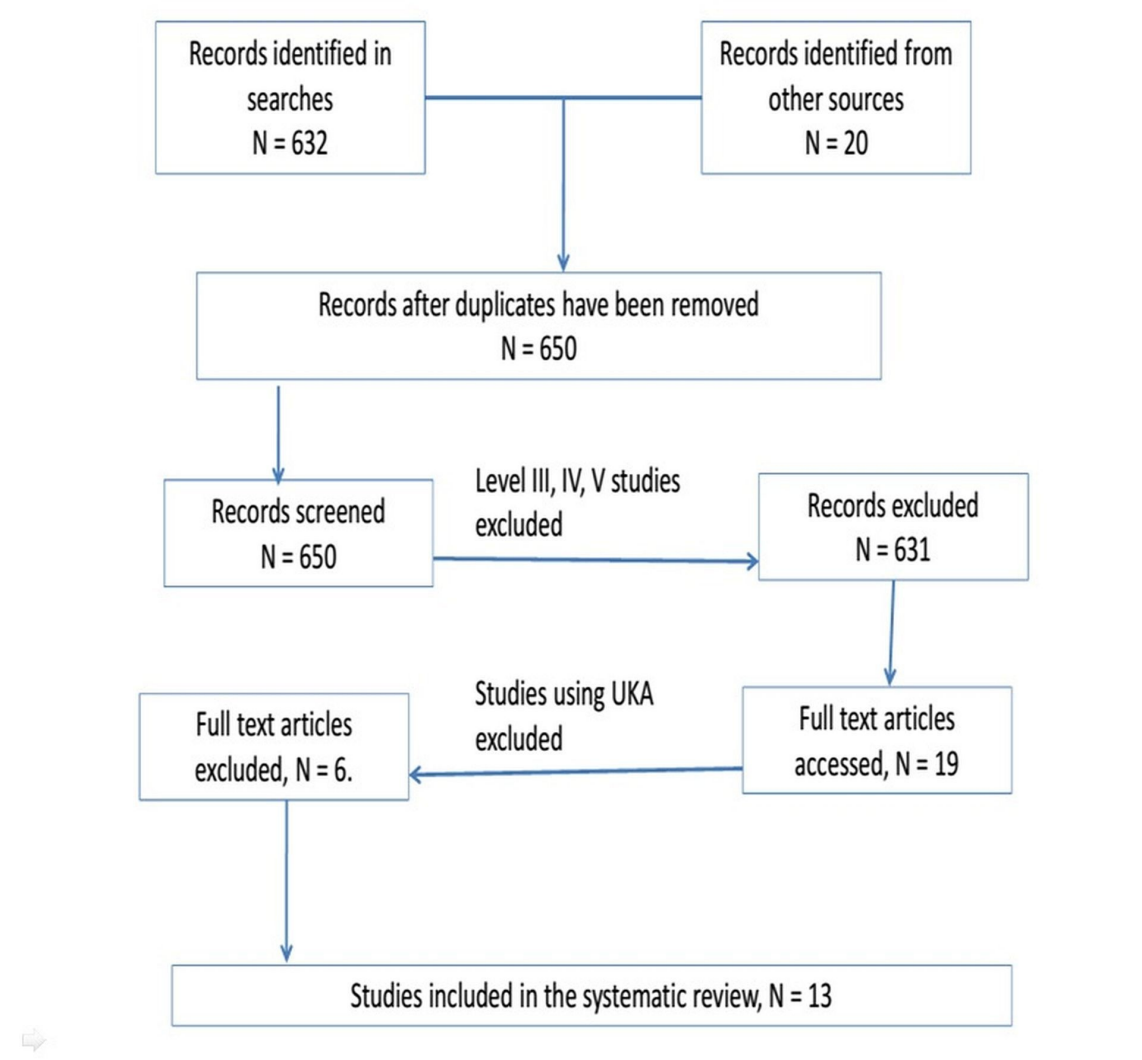
PRISMA Flow Diagram

The search identified 652 studies (632 from the online data search and 20 from references within the initial articles). After excluding duplicates and Level III, IV, and V studies, 19 articles remained. Further, unicompartmental knee arthroplasty was excluded from the search results, which finally led to the exclusion of six studies. The remaining 13 studies, which included data for 5,159 TKRs performed in five countries, satisfied the inclusion and exclusion criteria for this systematic review (Figure 1).

It is not clear from any of these studies if any bilateral simultaneous TKR cases were included, although 10 studies have remarked on the inclusion of bilateral staged TKR procedures [12-14, 16-17, 19-21, 23-24]. The included studies all had patient groups which were matched for age, gender, and preoperative clinical scores. A summary of included studies is noted in Table 1.

**Table 1:** Outcomes as Reported in the Literature BMI = body mass index; DVT = deep vein thrombosis; IKS =  International Knee Score; KSS = Knee Society Score; OKS = Oxford Knee Score; SF-12 = 12-Item Short Form Health Survey

Author	Study Level	BMI Groups	n	Follow-up in Months	Outcomes Reported
Amin, et al. 2006 [12]	Level II	BMI <30, >40	82	Mean 44	Survival, Revision, Infection, DVT, Radiolucencies, KSS
Chesney, et al. 2008 [13]	Level II	BMI <30, >40	744	Mean 60	Infection
Dewan, et al. 2009 [14]	Level II	BMI <30, >40	126	Mean 64.8	Survival, Revision, Radiolucencies, KSS
Dowsey, et al. 2009 [15]	Level II	BMI <30, >40	622	Mean 12	Survival, Revision, Infection
Dowsey, et al. 2010 [16]	Level II	BMI <30, >40	268	Mean 12	Survival, Infection, DVT, IKS
Ersozlu, et al. 2008 [17]	Level II	BMI <30, >40	82	Mean 32	Revision, Infection, DVT, Radiolucencies, KSS
Krushell, et al. 2007 [18]	Level II	BMI <30, >40	78	Mean 60	Survival, Revision, Infection, DVT, Radiolucencies, KSS
Napier, et al. 2014 [19]	Level II	BMI <30, >40	100	Mean 12	Infection, DVT, OKS, SF-12
Rajgopal, et al. 2008 [20]	Level II	BMI <30, >40	550	Mean 12	WOMAC, SF-12
Winiarsky, et al. 1998 [21]	Level II	BMI <30, >40	1818	Mean 58	Survival, Infection, KSS
Issa, et al. 2013 [22]	Level II	BMI <30, >40	105	Mean 52	Survival, KSS
Spicer, et al. 2001 [23]	Level II	BMI <30, >40	484	Mean 48	Survival, Revision, Radiolucencies
Mont, et al [24]	Level II	BMI <30, >40	100	Mean 62	Survival, Revision, Infection, Radiolucencies

Of the 13 articles cited, all but two reported on knee scores on either or both the control (NMO) and the MO groups; six studies examined radiolucent lines [12, 14, 17-18, 23-24]. Eight studies reported on complications associated with obesity [12, 14, 16-19, 21, 24]. Seven studies reported on revision data in MO patients [12, 14, 17-19, 23-24].

Female patients comprised more than 50% of the total number of patients in all but one the cohorts under investigation that reported on the characteristics [12-24]. The majority of patients in the MO group were younger than the control subjects in seven of the studies reporting on the age of the respondents and controls [14-18, 21, 23]. All the studies reviewing the KSS reported a postoperative improvement in both the controls and subject MO cohorts [25]. Higher postoperative knee scores in the MO group as compared to the control NMO group were described in three studies [13, 19, 22] while changes in the mean knee scores favored the NMO groups [12, 16-18, 21, 23] in six studies in this analysis. One article employed the Western Ontario and McMaster Universities Osteoarthritis Index (WOMAC) [20]. One study used the Oxford Knee Score (OKS) [19]. The rest used the various components of the Knee Society Score (KSS). The NMO group in three studies had higher postoperative knee scores (Table 2) [12, 14, 16].  

**Table 2:** DVT as Reported in the Literature NMO = non-morbid obese, MO = morbid obese; NR = p-value not reported

Author	Number of Procedures	Deep Vein Thrombosis
	*NMO group vs MO group*	*NMO group vs MO group*
Amin, et al. 2006 [12]	41 : 41	0 % vs 9.75% (NR)
Dowsey, et al. 2010 [16]	211 : 57	0.47% vs 5.26% (p-value 0.018)
Ersozlu, et al. 2008 [17]	40 : 42	0% vs 0% (NR)
Krushell, et al. 2007 [18]	39 : 39	2.56% vs 2.56% (NR)
Napier, et al. 2014 [19]	50 : 50	0% vs 0% (NR)
Winiarsky, et al. 1998 [21]	1768 : 50	0% vs 0% (NR)

Only one of these studies reported a significantly higher change score for controls compared with MO: 71 versus 61 on the OKS, respectively (p-value < 0.001) [16]. One of the studies with a higher control postoperative score did not show a significant difference in the MO cohort (p > 0.05) [14]. Only four studies commented on the significance of the functional outcome findings [14, 16, 18, 21]. Of the remaining studies, two did not comment at all on changes in functional outcomes in the respective cohorts [13, 15].

The overall complication rate in the MO group was higher across seven of the eight studies (75%) that looked into complications [12, 14, 16-17, 19, 21, 24]. This difference was statistically significant in three studies cited [12, 16, 21]. None of the studies revealed mortality within 90 days of the index surgery. DVT was reported as a serious complication in six studies with four studies noting no significant differences between the NMO and the MO groups (Table 2) [12, 16-19, 21].

Superficial infection rates across the studies showed a tendency to more MO group infections compared to the NO group [12-13, 16-19, 21]. Six studies reported on the incidence of deep prosthetic infection [12-13, 17-19, 21]. One study results quoted the presence of deep infection in the control NMO but none in the MO group [19]. Three studies reported an increased incidence of deep infection in the MO group as compared to the NMO group [12-13, 21]. Two studies reported no difference in the incidence of deep infection between the NO and NMO groups [17-18]. No progression to deep infections was found in two studies that had reported on superficial infections (Table 3) [17-18].

**Table 3:** Infection Rates in the Literature NMO = Non-morbid obese; MO = Morbidly obese; NR = not reported

Author	Superficial Infection % NMO : MO Group	Deep Infection % NMO : MO Group
Amin, et al. [12]	0 vs 9.75% (NR)	0 vs 11.7% (NR)
Chesney, et al. [13] Separate superficial and deep infection rates not reported, overall infection rate p value >0.05	3.1% vs 8.8%	1.1% vs 2.9%
Dowsey, et al. [16]	2.8% vs 15.7% (p value < 0.05)	NR
Ersozlu, et al. [17]	2.38% vs 9.52% (NR)	0% vs 0%
Krushell, et al. [18]	0 vs 20.5 % (p value < 0.05)	0% vs 0%
Napier, et al. [19]	0% vs 8% (NR)	4% vs 0% (NR)
Winiarsky, et al. [21]	2% vs 22% (p value < 0.05)	0.6% vs 10% (p value < 0.05)
Mont, et al. [24]	2% vs 0% (NR)	NR

Radiolucent lines were examined in six studies with a higher number seen in the MO group as compared to the NMO group. Revision rates were reported in seven studies [12, 14, 17-19, 23-24]. The MO group had higher revision rates in four studies among the seven studies quoted [12, 18, 23-24]. No difference in revision rates between the two groups was reported in one study [17].

Of the studies that had survivorship data, all of them except one showed good to excellent prosthesis survivorship in the MO group as well as in the NMO control group. Information in the MO group was missing in one study with six studies not having reported on the same [21].

The studies included in the systematic review were assessed for the difference between the NMO and MO groups. The mean BMI of the different studies for the NO group was 27.02 as compared to the mean BMI of 44.64 in the MO group.

**Figure 2:**
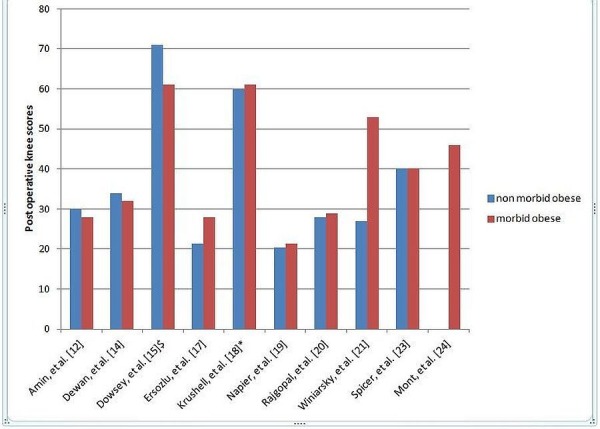
Postoperative Knee Scores in the Literature $ = IKS (International Knee Score); * = WOMAC (Western Ontario and McMaster Universities Arthritis Index)

The mean change of KKS in the NMO group was 41.68 as compared to the mean change in KSS in the MO group of 44.58 (Figure 2).

The incidence of superficial infections in the MO group was higher than the NMO group. The mean incidence of the superficial infection rates were 12.69 in the MO group as compared to the overall incidence of 1.53 in the NMO group.

On assessing the mean incidence of deep infection rates in the MO group with the non-obese group, there was no statistically significant difference.

The overall complication rates in the MO group were higher than in the NMO group. The reported mean complication rates in the NMO group were 8.34 as compared to a mean incidence of 22.48 in the MO group.

## Discussion

TKR in the obese patient has been associated with some concerns, such as increased perioperative risks and implant longevity. Whenever it is offered to the MO group of patients, it is based on the assumption that these patients would be benefitted with this surgery. The synthesis of recent outcome data in robust Level I and Level II clinical studies is essential for clinicians and relevant to patients in their decision-making process. Hence, we aimed to look at the available data to assist clinicians and patients make informed decisions as to the risks and benefits of planned TKR in the MO population. A key aim of accurately reviewing matched cohort studies was to control for other risk factors and focus on the impact MO (as an independent risk factor) has on outcomes in primary TKR.

The prevalence of obesity has been quoted to be 35% in the general American population with morbid obesity (BMI > 40) also growing considerably from 2.9% to 6.3% as per the latest figures available [1]. In certain subgroups in the USA, the morbidly obese trend in non-Hispanic black females has been quoted to approach 20% in the 40- to 60-year age bracket. Obese patients comprise 52.1% of the total joint arthroplasty patients and are projected to increase in the years ahead [2]. Comparable trends have been extrapolated worldwide [3]. Our systematic review looked at studies where MO was compared to NMO strictly as per the WHO definition of obesity and its subgrouping [4].

Functional outcome scores after TKR do not vary remarkably between the majority of the NMO and MO groups in the short-term (Table 1).

Two studies reported significantly reduced functional scores for the MO group, but no significant change was reported between postoperative knee scores measured at six to 60 months and five to 14 years, respectively [12, 18]. More recent studies report no difference between the functional outcomes of the MO group compared to the NMO group [19-20, 23]. The MO clusters in the review had lower preoperative functional scores, and though the postoperative scores were lower than in the NMO group, the mean improvement was not significantly worse. The studies looking at short-term functional scores under 12 months’ follow-up revealed that there were no meaningful changes in implant survivorship at one year [15-16, 20]. It has led to an interest in assessing the effect MO has on the mid- to long-term picture in primary TKR.

**Figure 3:**
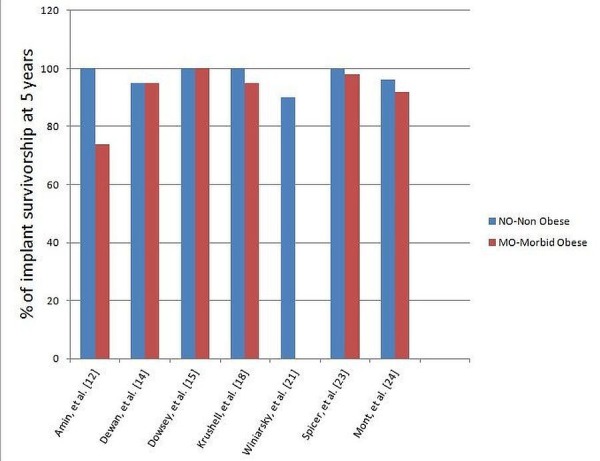
Revision Rates in Percentages as Reported in the Literature.

Foran, et al. assessed the obese (BMI: 30 to 45) and non-obese groups (BMI: 21 to 29) in a longer time frame [26]. The survival rate for the NMO group was appreciably worse than in the obese group with 33% in the obese group requiring a polyethylene spacer revision versus 60% of the non-obese knees (p = 0.069) at 14 years. The Kaplan-Meier plot for the obese group revealed a 71% (95% CI, 41.8 to 89.3) chance of survival at 176 months as compared to the non-obese group who had a 45.8% (95% CI, 21.5 to 72.2) chance of survival in the same time frame. Some studies have proposed this could be due to the relatively sedentary lifestyle of the MO and that this has a role to play in the reduction of pain, longer improved functional scores, and survivorship of the prosthesis. Younger and non-obese patients are assumed to lead a more active lifestyle leading to more polyethylene wear and subsequent revision. Functional improvement in postoperative status occurred in every group. The MO group reported lower satisfaction and functional rates as compared to the NMO group. Interestingly, contrary to common belief that obesity would worsen implant survival, revision rates were not increased in the MO group. Rajgopal, et al. showed greater improvement in function occurred in the MO group compared with non-morbidly obese patients (Figure 3) [20].

With regards to superficial infection, the morbidly obese fared poorer after TKR in all but one of the studies reporting on superficial infection [12-13, 16-19, 21]. This fact was further authenticated by the statistical analysis of the studies included in this meta-analysis. The morbidly obese group had a statistically significant difference in the rates of superficial infection rates. It changed when the studies were assessed for the incidence of low infection rates. The deep infection rates were not statistically significant between the NMO and MO groups in the present meta-analysis. The MO patients do have a tendency to develop more complications with regards to superficial infections. Plausible explanations range from inadequate macrophage recruitment to comorbidities. Two study results showed that that the number of monocytes that differentiated into macrophages was lower in obese subjects than it was in healthy subjects [27-28]. The generation of a lymphocytic migration-inhibiting factor with normal levels of glucose was significantly less in obese subjects than it was in controls [27] (p < 0.001). Obesity also has been regularly linked with hyperglycemia and insulin resistance, which has a detrimental action on leukocyte recruitment [27]. Low intraoperative subcutaneous tissue oxygen tension in the MO group has been associated with poor surgical outcomes [29]. Superficial infections, when managed well, usually do progress to complete healing with no long-lasting impact on the results. Superficial infections did not always translate to deep infections, as we hypothesized, due to prompt wound care by the surgical team. Winiarsky, et al. reported that, with better awareness of this potential complication, better wound closure and attention in the MO group resulted in no infection or wound complication observed in the last four years of the study [21].

**Figure 4:**
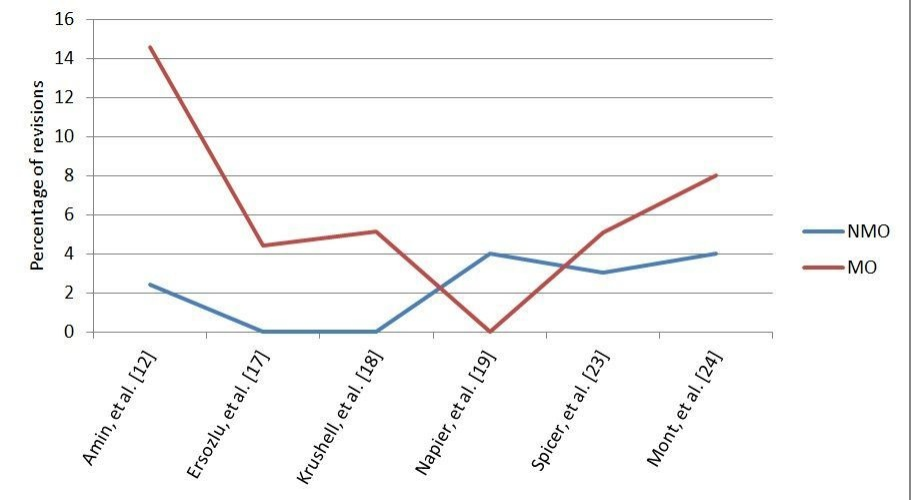
Implant Survivorship in Five Years.

Most earlier studies that examined the relationship between obesity and TKR did not address the issue of revision rates. The studies which have investigated the implant survivorship have shown mixed results. Amin, et al. reported inferior implant survivorship in the MO group as compared to the NMO group [12]. Spicer, et al. reported a higher incidence of radiolucency and osteolysis but that did not result in a statistically significant difference in the revision rates [23]. Dewan, et al. reported the much higher incidence of patellar translucencies in the MO group but reported overall similar outcomes between the NMO and MO group [12]. They suggested that the patients who develop patellar translucencies should be followed up regularly as they may have higher rates of complications. The reason for similar revision rates in the NMO and MO group, even in the presence of increased incidence of radiolucencies, can be the reduced activity levels in the MO group. Revisions occurred more often in the obese patients in other studies as well (Figure 4) [30].

Newer studies propose that it may not be the BMI index that has a significant role to play in implant survivorship but rather the absolute body weight [23]. The major limitations of most of the studies reporting implant survivorship are the comparatively short-term follow-ups. Mortality and major morbidities, such as thromboembolic events, are major concerns but are extremely low after TKR in the studies assessed. The overall complication rates in the morbidly obese groups were significantly higher than in the non-obese group after TKR (p < 0.05). This underlines the need for careful selection and screening of the morbidly obese patients before the TKR is performed.

No mortality was reported in any of the studies in the first 90 days following the surgery, and hence, it can be assumed that in carefully selected MO patients, TKR surgery may be considered a safe procedure. In only one early study was the rate of DVT higher than the rest [12]. The small incidence of the main complications in patients undergoing TKA suggests that the old medical clearance process works well in optimizing patients with comorbidities. Standardized DVT prophylaxis has led to the reduction of such complications across all groups.

## Conclusions

In doing our study, various factors prevented us from summarizing the research and providing combined point assessments, such as variations on the type of functional outcome scores used or the categorization of BMI. Secondly, not all the studies took into account key variables, such as the extent of obesity, mean BMI in each group, infection rates, the presence of radiolucencies, or revision rates. Some studies lacked a consistent definition specifying important postoperative adverse events or arthroplasty complications in each subgroup examined. Finally, many of the studies identified initially in the literature search were based on databases and registry data and had to be excluded.

As far as the implant survivorship is concerned in the MO group, studies with longer-term follow-up are needed. Many studies have shown increased incidence of radiolucencies and osteolysis in the MO group, but this did not always translate into higher revision rates, possibly due to decreased activity levels in the MO group. There is a need for the MO patients to be in close follow-up for monitoring the progression of radiolucencies and any further related complications. It is still debatable whether simultaneous bilateral TKR is a safe procedure to offer to the morbidly obese. None of the studies we assessed have reported on including simultaneous bilateral TKR cases in their studies. Some of the authors have reported doing bilateral TKR in the morbidly obese but have not mentioned having done these as a simultaneous procedure. Chesney, et al. reported on 160 bilateral cases, Dewan, et al. reported on 31 patients undergoing staged bilateral procedures, and Ersozlu, et al. specifically did a matched study that involved all the arms of his study having had staged bilateral TKR at four to 11 days apart. The outcomes reported no revisions, and there was no significant variation (p > 0.05) in the success rates between the NO and the MO groups at the two-year follow-up mark. This is an area that will require more research to assess for functional outcomes in the simultaneous bilateral TKRs. 
